# An Efficient Prephenate Dehydrogenase Gene for the Biosynthesis of L-tyrosine: Gene Mining, Sequence Analysis, and Expression Optimization

**DOI:** 10.3390/foods12163084

**Published:** 2023-08-17

**Authors:** Anying Ji, Pengfei Bao, Aimin Ma, Xuetuan Wei

**Affiliations:** 1 State Key Laboratory of Agricultural Microbiology, Huazhong Agricultural University, Wuhan 430070, China; anyingji@webmail.hzau.edu.cn (A.J.); baopengfei@webmail.hzau.edu.cn (P.B.); aiminma@mail.hzau.edu.cn (A.M.); 2 Shenzhen Institute of Nutrition and Health, Huazhong Agricultural University, Wuhan 430070, China; 3 Shenzhen Branch, Guangdong Laboratory for Lingnan Modern Agriculture, Genome Analysis Laboratory of the Ministry of Agriculture, Agricultural Genomics Institute at Shenzhen, Chinese Academy of Agricultural Sciences, Shenzhen 518000, China

**Keywords:** *Bacillus amyloliquefaciens*, L-tyrosine, prephenate dehydrogenase, recombinant expression, sequence analysis, element regulation

## Abstract

L-tyrosine is a key precursor for synthesis of various functional substances, but the microbial production of L-tyrosine faces huge challenges. The development of new microbial chassis cell and gene resource is especially important for the biosynthesis of L-tyrosine. In this study, the optimal host strain *Bacillus amyloliquefaciens* HZ-12 was firstly selected by detecting the production capacity of L-tyrosine. Subsequently, the recombinant expression of 15 prephenate dehydrogenase genes led to the discovery of the best gene, *Bao*-*tyrA* from *B. amyloliquefaciens* HZ-12. After the overexpression of *Bao*-*tyrA*, the L-tyrosine yield of the recombinant strain HZ/P43-*Bao*-*tyrA* reach 411 mg/L, increased by 42% compared with the control strain (HZ/pHY300PLK). Moreover, the nucleic acid sequence and deduced amino acid sequence of the gene *Bao*-*tyrA* were analyzed, and their conservative sites and catalytic mechanisms were proposed. Finally, the expression of *Bao*-*tyrA* was regulated through a promoter and 5′-UTR sequence to obtain the optimal expression elements. Thereby, the maximum L-tyrosine yield of 475 mg/L was obtained from HZ/P43-UTR3-*Bao*-*tyrA*. *B. amyloliquefaciens* was applied for the first time to produce L-tyrosine, and the optimal prephenate dehydrogenase gene *Bao*-*tyrA* and corresponding expression elements were obtained. This study provides new microbial host and gene resource for the construction of efficient L-tyrosine chassis cells, and also lays a solid foundation for the production of various functional tyrosine derivatives.

## 1. Introduction

The aromatic amino acid L-tyrosine is a nutritionally essential amino acid for humans, and it has been widely used in food additives, dietary supplements, pharmaceuticals, and chemicals [1,2,3]. Moreover, L-tyrosine is a common precursor for the synthesis of various high-value-added natural active substances such as salvianic acid A, resveratrol, caffeic acid, hydroxytyrosol, salidroside, curcumins, benzylisoquinoline alkaloids (BIAs), and so on [4,5,6,7,8,9]. These derivatives are endowed with various functions, such as antioxidant, anticoagulation and anti-inflammatory activities [10,11]. Therefore, L-tyrosine has prospects for broad application as a platform compound, and the demand for nutritional chemicals derived from L-tyrosine is constantly increasing [12,13,14,15]. For example, the market-sharing of the common L-tyrosine derivivates of flavonoids will reach 1.26 billion USD by 2026 according to market research reports [16].

Most nutritional chemicals are extracted from plants, and low extraction efficiency cannot adequately meet the growing demand for these naturally active substances [17,18]. Chemical synthesis methods are also applied for nutritional chemicals. However, chemical synthesis processes usually cause problems of environmental pollution, high energy consumption, the generation of different toxic intermediates, and the formation of unstable intermediates, which can lead to a noticeable decrease in the desired chemical productivity and economic yield [16]. Fortunately, green and sustainable biosynthesis methods can provide an alternative strategy for producing valuable chemicals in an environmentally friendly manner [19,20]. These sustainable biosynthesis methods also present the advantages of shorter process cycles, higher efficiency, and simpler extraction processes [21]. At present, the synthesis of various high-value functional nutritional chemicals, such as salvianic acid A and (2S)-naringenin have been achieved [22,23]. An alternative way to produce nutrient chemicals is to develop chassis cells with high yield of L-tyrosine and then introduce different synthesis modules, which results in the synthesis of natural active substances [5,8]. Therefore, the construction of efficient L-tyrosine chassis cells is important for the production of nutritional chemicals derived from L-tyrosine.

Although L-tyrosine has broad applications in the market, the excessive production of L-tyrosine by microorganisms has been a huge challenge to overcome due to the complex gene regulation [24,25]. At present, the L-tyrosine synthesis pathway has been obtained for most microorganisms [16,21,26]. The formation of 4-hydroxyphenylpyruvate from prephenate through oxidative decarboxylation is an important reaction for the synthesis of L-tyrosine [27,28,29]. The dehydrogenases specially used for L-tyrosine biosynthesis are the TyrA protein family, including prephenate dehydrogenase, cyclohexadienyl dehydrogenase, and aromatic acid dehydrogenase [30,31,32]. At present, the prephenate dehydrogenase is used in the production of L-tyrosine in several microorganisms by heterologous expression, such as *Escherichia coli*, *Saccharomyces cerevisiae*, *Bacillus licheniformis*, etc. [3,24,33]. Xu et al. overexpressed the gene *tyrA* from *B. licheniformis*, indicating that the expression level of *tyrA* plays an important role in improving the production of L-tyrosine [34].

The main microorganism currently used to produce L-tyrosine is *E. coli*, and the existence of endotoxins leads to it being difficult to apply in the food industry [35]. Compared to other microorganisms, *Bacillus* has become an alternative strain [36]. *Bacillus* is a class of Gram-positive bacteria widely existing in nature, which can produce spores in harsh environments. In addition, *Bacillus* species have the advantages of easy cultivation, clear inherited backgrounds, convenient gene modification, short fermentation cycle, and robustness in industrial fermentation [37]. These characteristics have caused *Bacillus* species to be the preferred organisms for the industrial production of various products, such as platform chemicals. Thereby, *Bacillus subtilis*, *B. licheniformis*, and *B. amyloliquefaciens* have been designated Generally Recognized As Safe (GRAS) strains by the US Food and Drug Administration (FDA) [37], and they have been widely used in the production of nutritional chemicals, such as riboflavin [38], spermidine [39], S-adenosylmethionine [40], N-acetylglucosamine, N-acetylneuraminic acid, chondroitin, poly-gamma-glutamic acid, and so on [41]. Additionally, *Bacillus* species can produce a wide range of products after genetic modification using novel synthetic biology strategies and efficient genetic engineering tools [42]. Therefore, it is believed that the production of L-tyrosine by *Bacillus* species is feasible and of great significance.

In this study, a series of food-safe *Bacillus* strains were evaluated to select the optimal host strain for L-tyrosine production, and the recombinant expression of 15 prephenate dehydrogenase-related genes was carried out. The optimal prephenate dehydrogenase gene and corresponding expression element suitable for the host strain were obtained, and its catalytic mechanism was further clarified. L-tyrosine is a key precursor for the synthesis of various functional substances. This study can provide new *Bacillus* host and gene resources for the construction of efficient L-tyrosine chassis cells, which can laid a solid foundation for production of various bioactive derivatives from tyrosine.

## 2. Materials and Methods

### 2.1. Strains and Plasmids

All the constructed strains and plasmids in the present study are shown in Table 1. The *B. amyloliquefaciens*-engineered strains were constructed based on the native strain of *B. amyloliquefaciens* HZ-12. All expression vectors derived from pHY300PLK were constructed and prepared using *E. coli* DH5α as the host strain. The corresponding primers used in the present study are shown in Table S1 in the Supplementary Materials section.

### 2.2. Recombinant Expression of the Prephenate Dehydrogenase Gene tyrA

The cloned prephenate dehydrogenase gene was identified using heterologous expression in *B. amyloliquefaciens* HZ-12 following the procedure reported in our previous study. Thereby, taking the gene *Bao*-*tyrA* expression strain as an example, the gene fragment of *Bao*-*tyrA* was amplified with a pair of primers, *Bao*-*tyrA*-F and *Bao*-*tyrA*-R, using the *B. amyloliquefaciens* HZ-12 DNA template, and then the gene *Bao*-*tyrA* was fused with the P43 promoter amplified from *B. subtilis* 168 and the T*amyL* terminator obtained from *B. licheniformis* WX-02 using overlap extension PCR (SOE-PCR). After digestion of fusion fragment and pHY300PLK plasmid by restriction enzymes of *Bam*HI and *Xba*I, they were ligated to obtain the expression plasmid pHY-P43-*Bao*-*tyrA*. Finally, this expression plasmid was electro-transformed into the competent cells of *B. amyloliquefaciens* HZ-12, generating the recombinant strain HZ/P43-*Bao*-*tyrA*. Other recombinant strains were obtained using the same procedure in this study. Moreover, synthetic 5′-UTRs were designed using UTR Library Designer (http://sbi.postech.ac.kr/utr_library, accessed on 6 July 2022) to fine-tune gene expression levels [43].

### 2.3. Chemicals

In this study, the TransStartFastPfu DNA polymerase and TransStartR easyTaq DNA polymerase were purchased from TransGen Biotech Co., Ltd. (Beijing, China). DNA restriction enzymes, T4 ligase, dNTPs, RNase, and DL5000 Marker were provided by Takara Biotechnology Co., Ltd. (Dalian, China). The DNA recovery kit and plasmid extraction kit were bought from Omega Bio-Tek, Guangzhou, China. Other chemicals were bought from Sinopharm Chemical Reagent Co., Ltd. (Shanghai, China).

### 2.4. Determination of L-tyrosine

To measure L-tyrosine, 1 mL of fermentation broth was vortexed with 0.6 mL of 1 M HCl for 1 h, and centrifuged at 10,000× *g* for 3 min. The supernatant was then filtered using a 0.22 μm membrane. The HPLC analysis conditions are listed as follows: Agilent 1100 HPLC chromatograph, ZORBAX Eclipse XDB-C18 (4.6 mm × 250 mm, 5 μm) column, mobile phase of 10% methanol, 90% sodium acetate (100 mM, pH 4.0), flow rate 0.6 mL/min, injection volume 10 μL, column temperature 30 °C, and UV detector with a detection wavelength of 280 nm. The L-tyrosine standard was used to calculate the concentration.

### 2.5. L-tyrosine Fermentation

The bacteria cells were transferred into 50 mL LB liquid medium containing 10 g/L tryptone, 5 g/L yeast extract, and 10 g/L NaCl. After culture at 37 °C and 180 rpm for 12 h, the seed cultures were obtained. Then, 3% (*v*/*v*) seed cultures were inoculated into the 50 mL L-tyrosine fermentation medium containing 22 g/L glucose, 3 g/L (NH_4_)_2_SO_4_, 6.75 g/L K_2_HPO_4_, 1.25 g/L KH_2_PO_4_, 1.5 g/L MgSO_4_·7H_2_O, 5 g/L sodium citrate, 3 g/L peptone, 6 g/L yeast extract, 4 g/L FeSO_4_·7H_2_O, 4 g/L CaCl_2_, 1 g/L MnSO_4_·5H_2_O, 0.4 g/L CoCl_2_·6H_2_O, 0.2 g/L ZnSO_4_·7H_2_O, 0.1 g/L AlCl_3_·6H_2_O, 0.1 g/L CuCl_2_·H_2_O, and 0.05 g/L H_3_BO_4_, cultured at 37 °C and 180 rpm for 36 h. When necessary, tetracycline was added at the final concentrations of 20 μg/mL.

### 2.6. Statistical Analysis

Each fermentation experiment was carried out in three independent replicates. SPSS 20.0 (IBM, Armonk, NY, USA) was used to calculate the means and standard deviations, and observe the significance. GraphPad Prism 8 (GraphPad Software Inc., San Diego, CA, USA) was applied to deal with the data and plot the graphs.

## 3. Results and Discussion

### 3.1. Screening the Optimal Host Strain for the Synthesis of the Platform Compound L-tyrosine

**Figure 1:**
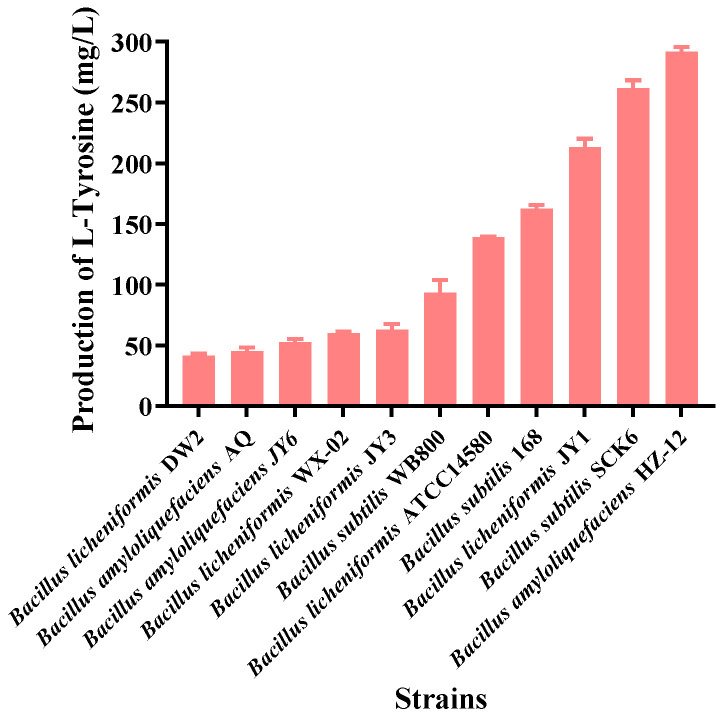
Evaluation of L-tyrosine production by serial host strains.

At present, the host strains for the production of platform compound L-tyrosine by metabolic engineering are concentrated in *E. coli* [26,44]. Juminaga et al. constructed a modular biosynthesis pathway in *E. coli* MG1655, and each module was optimized to achieve the optimal combination. Finally, the L-tyrosine yield reached 2.6 g/L, 79% of the maximum theoretical yield under corresponding fermentation conditions [26]. By using global transcription machinery engineering and high-throughput screening strategies, the rpoA mutant E. coli strains encoding RNA polymerase subunits were obtained, which could produce 13.8 g/L L-tyrosine in a 2 L fermenter [44]. However, the existence of endotoxins in *E. coli* hinders its applicability in the food industry [35]. In contrast, Bacillus species have become a promising alternative due to their advantages of their Generally Recognized As Safe (GRAS) status, good growth on cheap carbon sources, distinct endogenous metabolism, and robustness in industrial fermentations [34]. Hence, a series of Bacillus strains (shown in Table 1) stored in this laboratory were verified with L-tyrosine as the monitoring target for obtaining the optimal host strain. As shown in Figure 1, the L-tyrosine yield of the strain B. amyloliquefaciens HZ-12 reached 297 mg/L after fermentation for 36 h, much higher than other strains. B. amyloliquefaciens HZ-12 has a high initial L-tyrosine yield, and it has also been broadly applied in the biosynthesis of functional substances due to its genetic transformation potential, being able to transform into organic compounds such as spermidine and S-adenosylmethionine [39,40]. Therefore, the B. amyloliquefaciens HZ-12 was selected for engineering modification in the subsequent experimental operations.

### 3.2. Effects of Different Prephenate Dehydrogenase Genes on Biosynthesis of L-tyrosine

At present, the L-tyrosine biosynthesis pathways in *E.coli*, *Bacillus*, and other microorganisms have been analyzed, which has laid a certain theoretical foundation for the subsequent optimization of L-tyrosine production [16,26,33]. Prephenate dehydrogenase is a key pathway enzyme for the synthesis of L-tyrosine, and its activity affects the synthesis of metabolic end products [33,34]. Kim et al. overexpressed the mutant gene *tyrA* from *E.coli*, indicating that the expression level of *tyrA* plays an important role in improving the production of L-tyrosine [24]. Therefore, in order to obtain the optimal prephenate dehydrogenase gene, the gene of bifunctional chorismate mutase/prephenate dehydrogenase was amplified from *E. coli* MG1655, and other prephenate dehydrogenase genes were cloned from *Lactobacillus plantarum*, *S. cerevisiae*, *Corynebacterium glutamate*, *B. subtilis*, *B. licheniformis*, *Bacillus coagulans*, *Bacillus pumilus*, *Bacillus megaterium*, *Bacillus thuringiensis*, *Bacillus cereus*, and *B. amyloliquefaciens*. Moreover, we also cloned the cyclohexadiene dehydrogenase genes from *Zymomonas mobilis* and *Pseudomonas aeruginosa*, another type of catalytic enzyme of TyrA protein family [30]. The 15 obtained gene fragments were separately inserted into plasmid pHY300PLK to generate corresponding expression plasmids. Then, these expression plasmids were electrically transformed into *B. amyloliquefaciens* HZ-12 to obtain recombinant strains, including HZ/P43-*Bao*-*tyrA*, HZ/P43-*Eco*-*tyrA*, HZ/P43-*Lpt*-*tyrA*, HZ/P43-*Sce*-*tyrA*, HZ/P43-*Cgb*-*tyrA*, HZ/P43-*Bsu*-*tyrA*, HZ/P43-*Bld*-*tyrA*, HZ/P43-*Bcoa*-*tyrA*, HZ/P43-*Bpum*-*tyrA*, HZ/P43-*Bmh*1-*tyrA*, HZ/P43-*Bmh*2-*tyrA*, HZ/P43-*Btb*-*tyrA*, HZ/P43-*Bce*-*tyrA*, HZ/P43-*Zmc*-*tyrC*, and HZ/P43-*Pap*-*tyrA*.

**Figure 2:**
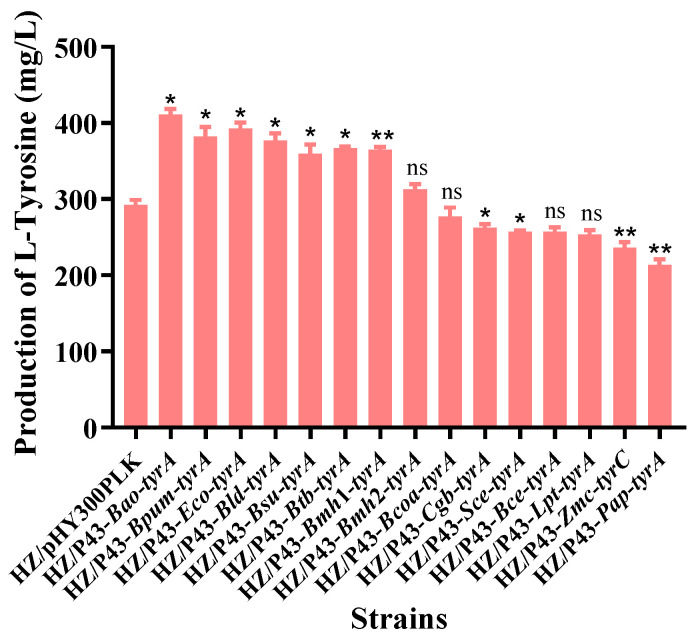
Effects of enhanced expression of the key enzyme genes *tyrA* from different species on L-tyrosine production. Note: * means significant difference (*p* < 0.05), ** means very significant difference (*p* < 0.01), and ns means no significant difference.

The L-tyrosine yields of all the 15 engineered strains were measured after shake-flask cultivation for 36 h under the same experimental conditions. As shown in Figure 2, the highest yield (411 mg/L) was obtained after the enhanced expression of the gene *tyrA* from *B. amyloliquefaciens* HZ-12, which was 42% higher than that of the control strain HZ/pHY300PLK. In addition, the expression of the gene from *E. coli*, *B. subtilis*, *B. licheniformis*, *B. pumilus*, *Bacillus megaterium*, *B. thuringiensis*, and *Bacillus cereus* also has a significant improvement effect on the synthesis of L-tyrosine. The data indicate that the efficient expression of the prephenate dehydrogenase gene is crucial for the synthesis of L-tyrosine, and various prephenate dehydrogenase gene resources suitable for L-tyrosine synthesis were obtained, especially the gene *Bao*-*tyrA* from *B. amyloliquefaciens* HZ-12.

### 3.3. Amino Acid Sequence Analysis and Possible Catalytic Mechanism

**Figure 3:**
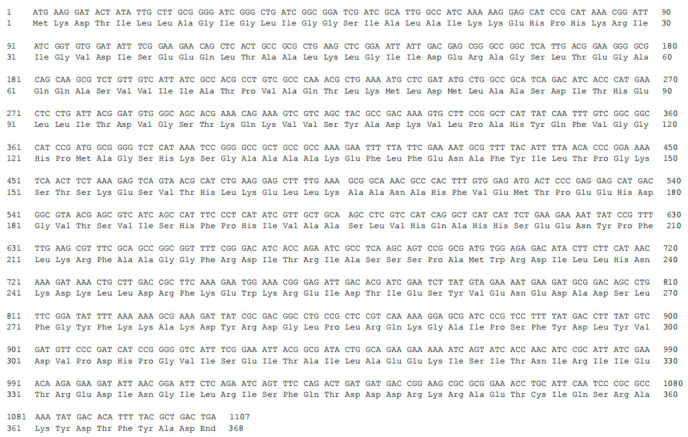
Gene sequence (**upper part**) and deduced amino acids sequence (**lower part**) of *Bao*-*tyrA*.

**Figure 4:**
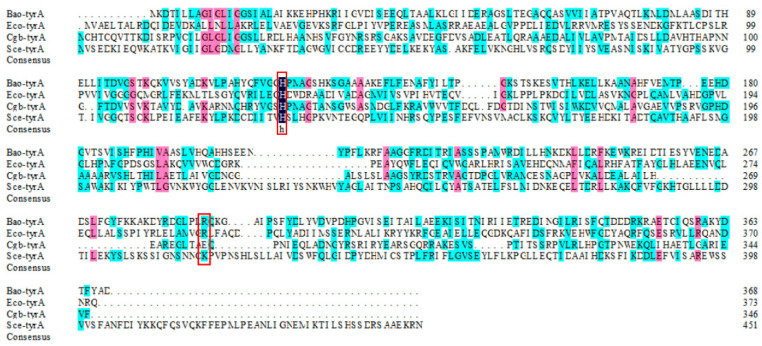
Comparison of amino acid sequences of the prephenate dehydrogenase gene *tyrA* from *B. amyloliquefaciens* and other species. The conserved active sites (H131 and R294) were marked in red box.

Prephenate dehydrogenase catalyzes the synthesis of p-hydroxyphenylpyruvate using prephenate and NAD^+^ as substrates [45]. To explain the function of the prephenate dehydrogenase gene *Bao-tyrA* from *B. amyloliquefaciens* HZ-12, the gene sequence (1107 bp) and corresponding amino acids were further analyzed. The gene sequence of *Bao*-*tyrA* was translated into a sequence containing 368 amino acids by using BioEdit v7.0.9.0 software (Figure 3). Then, the sequence was aligned with the previously reported amino acid sequences (Figure 4), and the similarities with *E. coli* (NC_000913), *C. glutamate* (CAF18797), and *S. cerevisiae* (NC_001134) were 20.89%, 25.86%, and no similarity, respectively. In addition, the TyrA protein from *E. coli* is a bi-functional enzyme that can display the activities of chorismate mutase/prephenate dehydrogenase, while the TyrA protein from *B. amyloliquefaciens* HZ-12 only shows the activity of prephenate dehydrogenase [34,46]. Sequence alignment revealed that these amino acid sequences contained the same conserved sites. Therefore, His131 might assist in the transfer of hydride from prephenate to NAD^+^ in the dehydrogenase reaction [45]. Arg294 might interact specifically with the cyclic carboxylate at C-1 of prephenate [47]. This further illustrates the catalytic mechanism of the prephenate dehydrogenase coded by the gene *Bao-tyrA* in *B. amyloliquefaciens* HZ-12.

### 3.4. Effects of Different Promoters of Bao-tyrA on Biosynthesis of L-tyrosine

**Figure 5:**
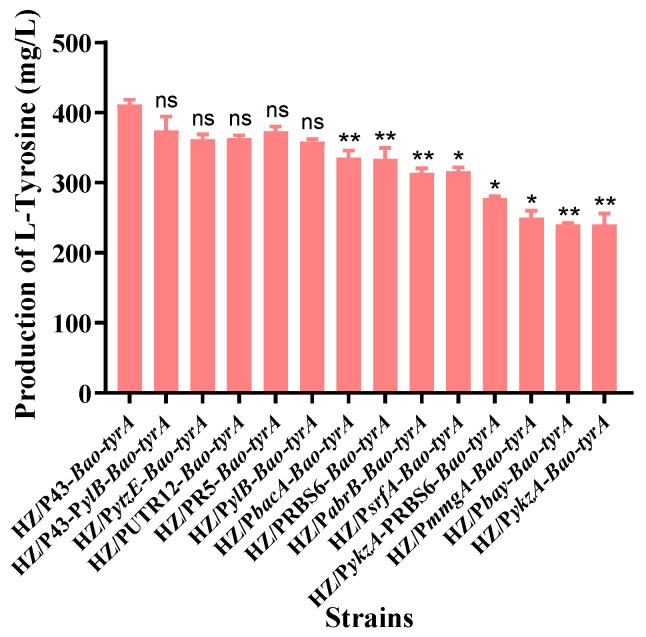
Effect of replacement of promoter of the gene *Bao-tyrA* on L-tyrosine production. Note: * means significant difference (*p* < 0.05), ** means very significant difference (*p* < 0.01), and ns means no significant difference.

The promoter is one of the key factors that can affect the gene expression level, and it constitutes an important genetic regulatory element in the complex framework of transcriptional control [48,49]. An efficient promoter, SPL-21, was screened from *Streptomyces* to control the expression of toyF, which significantly increased the production of toyocamycin [50]. A high-strength promoter, P*_tnrQ_*, was mined from *B. subtilis* based on transcriptome data, and it could double the amylase expression [51]. Based on genome-wide microarray analyses, a toolbox of novel promoters was obtained from *B. megaterium* to offer versatile promoter strength, and the final progesterone yields of 3.6 mM were found to be increased compared with the control promoter [52]. This indicates that efficient promoters can further improve the production of metabolites. Therefore, in order to further optimize the expression level of the gene *Bao*-*tyrA*, promoters with different strengths were used, and corresponding recombinant strains were obtained, including HZ/P*srfA*-*Bao*-*tyrA*, HZ/P*ytzE*-*Bao*-*tyrA*, HZ/P*ylb*-*Bao*-*tyrA*, HZ/P*bay*-*Bao*-*tyrA*, HZ/P*ykzA*-*Bao*-*tyrA*, HZ/P*ykzA*-PRBS6-*Bao*-*tyrA*, HZ/P*mmgA*-*Bao*-*tyrA*, HZ/P*abrB*-*Bao*-*tyrA*, HZ/P*bacA*-*Bao*-*tyrA*, HZ/PUTR12-*Bao*-*tyrA*, HZ/P43-P*ylb*-*Bao*-*tyrA*, HZ/PR5-*Bao*-*tyrA*, and HZ/PRBS6-*Bao*-*tyrA*. The L-tyrosine yields of all the 13 engineered strains were measured (Figure 5); it was found that different promoters had different effects on the expression of prephenate dehydrogenase. In comparison, the P43 promoter could drive the maximum L-tyrosine production in *B. amyloliquefaciens* HZ-12, while other promoters did not further increase L-tyrosine production. The data indicate that the constitutive promoter P43 has great advantages in the sustained and efficient expression of its regulated genes.

### 3.5. Redesign of the 5′-UTR of the Gene Bao-tyrA

**Figure 6:**
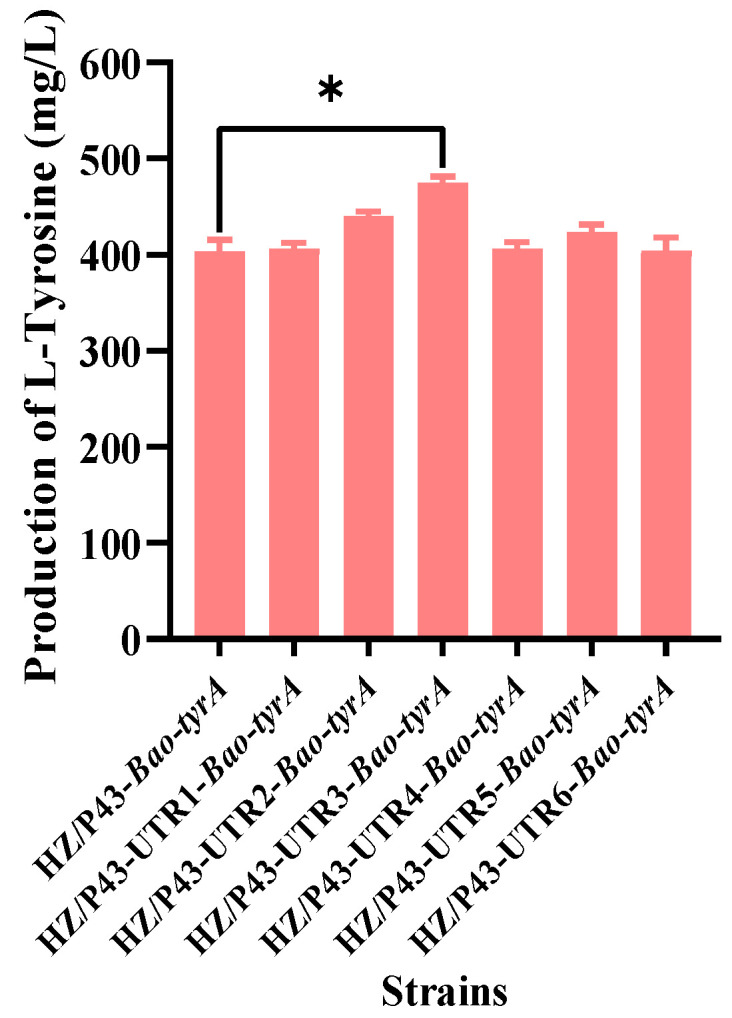
Effects of replacing 5′-UTR sequences of *Bao*-*tyrA* on L-tyrosine production. Note: * in the figure means significant difference (*p* < 0.05).

In addition to the promoter, 5′-UTR is also a factor affecting gene expression, and can play a role in post transcriptional regulation of genes [53,54]. Kim et al. introduced a constitutive promoter and a synthetic 5′-UTR into each gene of the L-tyrosine synthesis pathway, and then used UTR designer to control the expression level of PEP synthetase. After optimization, the L-tyrosine yield of *E. coli* was increased to 3.0 g/L [2]. Noh et al. introduced a strong inducible tac promoter with synthetic 5′-UTRs designed for high expression to the genes *hemA* and *hemL*, and the yield of 5-aminolevulinic acid was increased to 0.74 g/L after optimization [55]. This demonstrates that the 5′-UTR redesign is an effective strategy for optimizing the biosynthetic pathway. Therefore, to further optimize the expression of gene *Bao*-*tyrA* at the translation level, the five-terminal UTR sequence of gene *Bao*-*tyrA* was redesigned to precisely regulate gene expression. The corresponding gene-expression plasmids pHY-P43-UTR1-*Bao*-*tyrA*, pHY-P43-UTR2-*Bao*-*tyrA*, pHY-P43-UTR3-*Bao*-*tyrA*, pHY-P43-UTR4-*Bao*-*tyrA*, pHY-P43-UTR5-*Bao*-*tyrA*, and pHY-P43-UTR6-*Bao*-*tyrA* were constructed. These expression plasmids were then converted into *B. amyloliquefaciens* HZ-12, and corresponding recombinant strains were constructed, including HZ/P43-UTR1-*Bao*-*tyrA*, HZ/P43-UTR2-*Bao*-*tyrA*, HZ/P43-UTR3-*Bao*-*tyrA*, HZ/P43-UTR4-*Bao*-*tyrA*, HZ/P43-UTR5-*Bao*-*tyrA*, and HZ/P43-UTR6-*Bao*-*tyrA*. The L-tyrosine yields of all these six engineered strains after shake-flask cultivation for 36 h are shown in Figure 6. The redesigned UTR3 could significantly increase the L-tyrosine production to 475 mg/L, which was 16% higher than the control strain HZ/P43-*Bao*-*tyrA*. The data indicated that the redesign of the five-terminal UTR was a feasible strategy in the metabolic engineering of *Bacillus*.

The 5′-UTR redesign can be used to fine-tune the level of target gene expression within cells, and has been widely used in *E. coli* and *C*. *glutamate*. Lee et al. constructed a plug-in inhibitor expression library based on 5′-UTR redesign, and verified the expression range of the library. Subsequently, the library was applied to accurately control the throughput of key metabolic nodes in the synthesis pathway of target substances. Finally, the yield of 3-HP and lycopene reached 2.59 g/L and 11.66 mg/L, which, respectively, increased 16.5 times and 2.82 times compared with the parental strains [56]. Jiang et al. developed two plug-in repressor expression libraries by diversifying the translation levels of *phlF* and *mcbR* based on the 5′-UTR variants to accurately control the metabolic flux, which can reduce the production of L-lysine as a by-product and balance extracellular protein synthesis and cell growth. The two libraries resulted in a 28% and 12% increase in the production of Ectoine compared with the control strain, respectively [57]. This indicates that the redesign of 5′-UTR is important for the precise control of intracellular carbon flux, and can provide some guidance for the subsequent optimization of biosynthesis. However, few studies have been conducted to fine-tune the expression level of target genes in *B. amyloliquefaciens* by redesigning 5′-UTR sequences. This study fills in this gap and verifies that this strategy of redesigning 5′-UTR sequences can fine-tune the intracellular gene expression level in *B. amyloliquefaciens*, which can be further applied to the production of more target compounds.

## 4. Conclusions

This study obtained a food-safe strain, *B. amyloliquefaciens* HZ-12, with a high initial yield of L-tyrosine. A total of 15 prephenate dehydrogenase genes were successfully expressed to screen the optimal gene suitable for the host strain, and the gene *Bao-tyrA* from *B. amyloliquefaciens* HZ-12 was confirmed to be an efficient gene resource. In addition, promoter replacement and five-terminal UTR redesign were also carried out, and it was found that the use of a 5′-UTR-3 sequence driven by a P43 promoter could enhance the expression of prephenate dehydrogenase in *B. amyloliquefaciens*. This study provides new microbial and genetic resources for construction of a L-tyrosine chassis cell, which will be beneficial for metabolic engineering.

## Figures and Tables

**Table 1:** Strains and plasmids used in this study.

Strains or Plasmids	Characteristics	Source
*Bacillus amyloliquefaciens* HZ-12	Wild type	stored in lab
*Bacillus subtilis* 168	*trpC*2	stored in lab
*Bacillus subtilis* SCK6	Erm^R^, 1A751 derivate, *lacA*::P*_xylA_*-*comK*	stored in lab
*Bacillus subtilis* WB800	*nprE aprE epr bpr mpr*::*ble nprB*::*bsr* Δ*vpr wprA*::*hyg*	stored in lab
*Bacillus licheniformis* JY1	Wild type	stored in lab
*Bacillus licheniformis* JY3	Wild type	stored in lab
*Bacillus licheniformis* DW2	Wild type	stored in lab
*Bacillus licheniformis* WX-02	Wild type	stored in lab
*B. licheniformis* ATCC14580	Wild type	stored in lab
*B. amyloliquefaciens* JY6	Wild type	stored in lab
*B. amyloliquefaciens* AQ	Wild type	stored in lab
HZ/pHY300PLK	HZ-12 with pHY300PLK	this study
HZ/P43-*Eco*-*tyrA*	HZ-12 with pHY-P43-*Eco*-*tyrA*	this study
HZ/P43-*Bao*-*tyrA*	HZ-12 with pHY-P43-*Bao*-*tyrA*	this study
HZ/P43-*Bld*-*tyrA*	HZ-12 with pHY-P43-*Bld*-*tyrA*	this study
HZ/P43-*Bsu*-*tyrA*	HZ-12 with pHY-P43-*Bsu*-*tyrA*	this study
HZ/P43-*Btb*-*tyrA*	HZ-12 with pHY-P43-*Btb*-*tyrA*	this study
HZ/P43-*Bpum*-*tyrA*	HZ-12 with pHY-P43-*Bpum*-*tyrA*	this study
HZ/P43-*Cgb*-*tyrA*	HZ-12 with pHY-P43-*Cgb*-*tyrA*	this study
HZ/P43-*Sce*-*tyrA*	HZ-12 with pHY-P43-*Sce*-*tyrA*	this study
HZ/P43-*Lpt*-*tyrA*	HZ-12 with pHY-P43-*Lpt*-*tyrA*	this study
HZ/P43-*Bcoa*-*tyrA*	HZ-12 with pHY-P43-*Bcoa*-*tyrA*	this study
HZ/P43-*Bce*-*tyrA*	HZ-12 with pHY-P43-*Bce*-*tyrA*	this study
HZ/P43-*Bmh*1-*tyrA*	HZ-12 with pHY-P43-*Bmh*1-*tyrA*	this study
HZ/P43-*Bmh*2-*tyrA*	HZ-12 with pHY-P43-*Bmh*2-*tyrA*	this study
HZ/P43-*Zmc*-*tyrA*	HZ-12 with pHY-P43-*Zmc*-*tyrA*	this study
HZ/P43-*Pap*-*tyrA*	HZ-12 with pHY-P43-*Pap*-*tyrA*	this study
HZ/P*srfA*-*Bao*-*tyrA*	HZ-12 with pHY-P*srfA*-*Bao*-*tyrA*	this study
HZ/P*ytzE*-*Bao*-*tyrA*	HZ-12 with pHY-P*ytzE*-*Bao*-*tyrA*	this study
HZ/P*ylB*-*Bao*-*tyrA*	HZ-12 with pHY-P*ylB*-*Bao*-*tyrA*	this study
HZ/P*bay*-*Bao*-*tyrA*	HZ-12 with pHY-P*bay*-*Bao*-*tyrA*	this study
HZ/P*ykzA*-*Bao*-*tyrA*	HZ-12 with pHY-P*ykzA*-*Bao*-*tyrA*	this study
HZ/P*ykzA*-PRBS6-*Bao*-*tyrA*	HZ-12 with pHY-P*ykzA*-PRBS6-*Bao*-*tyrA*	this study
HZ/PmmgA-*Bao*-*tyrA*	HZ-12 with pHY-P*mmgA*-*Bao*-*tyrA*	this study
HZ/P*abrB*-*Bao*-*tyrA*	HZ-12 with pHY-P*abrB*-*Bao*-*tyrA*	this study
HZ/P*bacA*-*Bao*-*tyrA*	HZ-12 with pHY-PbacA-*Bao*-*tyrA*	this study
HZ/P12-*Bao*-*tyrA*	HZ-12 with pHY-P12-*Bao*-*tyrA*	this study
HZ/P43-P*ylB*-*Bao*-*tyrA*	HZ-12 with pHY-P43-ylB-*Bao*-*tyrA*	this study
HZ/PR5-*Bao*-*tyrA*	HZ-12 with pHY-PR5-*Bao*-*tyrA*	this study
HZ/PRBS6-*Bao*-*tyrA*	HZ-12 with pHY-PRBS6-*Bao*-*tyrA*	this study
HZ/P43-UTR1-*Bao*-*tyrA*	HZ-12 with pHY-P43-UTR1-*Bao*-*tyrA*	this study
HZ/P43-UTR2-*Bao*-*tyrA*	HZ-12 with pHY-P43-UTR2-*Bao*-*tyrA*	this study
HZ/P43-UTR3-*Bao*-*tyrA*	HZ-12 with pHY-P43-UTR3-*Bao*-*tyrA*	this study
HZ/P43-UTR4-*Bao*-*tyrA*	HZ-12 with pHY-P43-UTR4-*Bao*-*tyrA*	this study
HZ/P43-UTR5-*Bao*-*tyrA*	HZ-12 with pHY-P43-UTR5-*Bao*-*tyrA*	this study
HZ/P43-UTR6-*Bao*-*tyrA*	HZ-12 with pHY-P43-UTR6-*Bao*-*tyrA*	this study
pHY300PLK	*E. coli*–*Bacillus* shuttle vector for gene expression, Ap^r^, Tet^r^	stored in lab
pHY-P43-*Eco*-*tyrA*	pHY300PLK + P43 + *Eco*-*tyrA* + TamyL	this study
pHY-P43-*Bao*-*tyrA*	pHY300PLK + P43 + *Bao*-*tyrA* + TamyL	this study
pHY-P43-*Bld*-*tyrA*	pHY300PLK + P43 + *Bld*-*tyrA*+ TamyL	this study
pHY-P43-*Bsu*-*tyrA*	pHY300PLK + P43 + *Bsu*-*tyrA* + TamyL	this study
pHY-P43-*Btb*-*tyrA*	pHY300PLK + P43 + *Btb*-*tyrA* + TamyL	this study
pHY-P43-*Bpum*-*tyrA*	pHY300PLK + P43 + *Bpum*-*tyrA* + TamyL	this study
pHY-P43-*Cgb*-*tyrA*	pHY300PLK + P43 + *Cgb*-*tyrA* + TamyL	this study
pHY-P43-*Sce*-*tyrA*	pHY300PLK + P43 + *Sce*-*tyrA* + TamyL	this study
pHY-P43-*Lpt*-*tyrA*	pHY300PLK + P43 + *Lpt*-*tyrA* + TamyL	this study
pHY-P43-*Bcoa*-*tyrA*	pHY300PLK + P43 + *Bcoa*-*tyrA* + TamyL	this study
pHY-P43-*Bce*-*tyrA*	pHY300PLK + P43 + *Bce*-*tyrA* + TamyL	this study
pHY-P43-*Bmh*1-*tyrA*	pHY300PLK + P43 + *Bmh*1-*tyrA* + TamyL	this study
pHY-P43-*Bmh*2-*tyrA*	pHY300PLK + P43 + *Bmh*2-*tyrA* + TamyL	this study
pHY-P43-*Zmc*-*tyrA*	pHY300PLK + P43 + *Zmc*-*tyrA* + TamyL	this study
pHY-P43-*Pap*-*tyrA*	pHY300PLK + P43 + *Pap*-*tyrA* + TamyL	this study
pHY-P*srfA*-*Bao*-*tyrA*	pHY300PLK + P*srfA* + *Bao*-*tyrA* + TamyL	this study
pHY-P*ytzE*-*Bao*-*tyrA*	pHY300PLK + P*ytzE* + *Bao*-*tyrA* + TamyL	this study
pHY-P*ylB*-*Bao*-*tyrA*	pHY300PLK + P*ylB* + *Bao*-*tyrA* + TamyL	this study
pHY-P*bay*-*Bao*-*tyrA*	pHY300PLK + P*bay* + *Bao*-*tyrA* + TamyL	this study
HZ/P*ykzA*-*Bao*-*tyrA*	pHY300PLK + P*ykzA* + *Bao*-*tyrA* + TamyL	this study
pHY-P*ykzA*-RBS6-*Bao*-*tyrA*	pHY300PLK + PykzA-PRBS6 + *Bao*-*tyrA* + TamyL	this study
pHY-P*mmgA*-*Bao*-*tyrA*	pHY300PLK + P*mmgA* + *Bao*-*tyrA* + TamyL	this study
pHY-P*abrB*-*Bao*-*tyrA*	pHY300PLK + P*abrB* + *Bao*-*tyrA* + TamyL	this study
pHY-P*bacA*-*Bao*-*tyrA*	pHY300PLK + P*bacA* + *Bao*-*tyrA* + TamyL	this study
pHY-P12-*Bao*-*tyrA*	pHY300PLK + P12 + *Bao*-*tyrA* + TamyL	this study
pHY-P43-ylB-*Bao*-*tyrA*	pHY300PLK + P43-P*ylB* + *Bao*-*tyrA* + TamyL	this study
pHY-PR5-*Bao*-*tyrA*	pHY300PLK + PR5 + *Bao*-*tyrA* + TamyL	this study
pHY-PRBS6-*Bao*-*tyrA*	pHY300PLK + PRBS6 + *Bao*-*tyrA* + TamyL	this study
pHY-P43-UTR1-*Bao*-*tyrA*	pHY300PLK + P43 + UTR1-*Bao*-*tyrA* + TamyL	this study
pHY-P43-UTR2-*Bao*-*tyrA*	pHY300PLK + P43 + UTR2-*Bao*-*tyrA* + TamyL	this study
pHY-P43-UTR3-*Bao*-*tyrA*	pHY300PLK + P43 + UTR3-*Bao*-*tyrA* + TamyL	this study
pHY-P43-UTR4-*Bao*-*tyrA*	pHY300PLK + P43 + UTR4-*Bao*-*tyrA* + TamyL	this study
pHY-P43-UTR5-*Bao*-*tyrA*	pHY300PLK + P43 + UTR5-*Bao*-*tyrA* + TamyL	this study
pHY-P43-UTR6-*Bao*-*tyrA*	pHY300PLK + P43 + UTR6-*Bao*-*tyrA* + TamyL	this study

## Data Availability

The data used to support the findings of this study are available from the corresponding author upon request.

## Supplementary Materials

The following supporting information can be downloaded at: https://www.mdpi.com/article/10.3390/foods12163084/s1, Table S1: Primers used in this study.
