# Supplementary material for: An Efficient Prephenate Dehydrogenase Gene for the Biosynthesis of L-tyrosine: Gene Mining, Sequence Analysis, and Expression Optimization
Source: Foods. 2023 Aug 17;12(16):3084. doi: 10.3390/foods12163084 (PMC10453860; doi:10.3390/foods12163084)
Supplement: Supplementary file 1 [file foods-12-03084-s001.zip › foods-2525474-supplementary.pdf]

Table S1. Primers used in this study.

Primer name	Sequence of primer (5' to 3')
pHY300-F	GTTTATTATCCATACCCTTAC
pHY300-R	CAGATTTCGTGATGCTTGTC
P43-*Bao-tyrA*-F	CG**GGATCC**TGATAGGTGGTATGTTTTCGC
P43-*Bao-tyrA*-R	CAAGCAATATTGTGTCCTTCATGTGTACATTCCTCTCTTACCTATAA
Bao-tyrA-F	TTATAGGTAAGAGAGGAATGTACACATGAAGGACACAATATTGCTTG
Bao-tyrA-R	AATCCGTCCTCTCTGCTCTTTCAGTCAGCGTAAAATGTTTCA
TamyL-*Bao-tyrA*-F	TGAAACATTTTACGCTGACTGAAAGAGCAGAGAGGACGGATT
TamyL-*Bao-tyrA*-R	GC**TCTAGA**CGCAATAATGCCGTCG
P43-*Eco-tyrA*-F	CG**GAATTC**TGATAGGTGGTATGTTTTCGC
P43-Eco-tyrA-R	CGGTCAATTCAGCAACCATGTGTACATTCCTCTCTTACCTATAA
Eco-tyrA-F	TTATAGGTAAGAGAGGAATGTACACATGGTTGCTGAATTGACCG
Eco-tyrA-R	AATCCGTCCTCTCTGCTCTTTTACTGGCGATTGTCATTCG
TamyL-*Eco-tyrA*-F	CGAATGACAATCGCCAGTAAAAGAGCAGAGAGGACGGATT
TamyL-*Eco-tyrA*-R	GC**TCTAGA**CGCAATAATGCCGTCG
P43-*Bld-tyrA*-F	CG**GGATCC**TGATAGGTGGTATGTTTTCGC
P43-*Bld-tyrA*-R	CGTTTCATTCGGTTGAGTCATGTGTACATTCCTCTCTTACCTATAA
Bld-tyrA-F	TTATAGGTAAGAGAGGAATGTACACATGACTCAACCGAATGAAACG
Bld-tyrA-R	AATCCGTCCTCTCTGCTCTTTCAATCAGCATAAAATGTTTCG
TamyL-*Bld-tyrA*-F	CGAAACATTTTATGCTGATTGAAAGAGCAGAGAGGACGGATT
TamyL-*Bld-tyrA*-R	GC**TCTAGA**CGCAATAATGCCGTCG
P43-*Bsu-tyrA*-F	CG**GGATCC**TGATAGGTGGTATGTTTTCGC
P43-*Bsu-tyrA*-R	ATATTGTATCTTTCATTTGATTCATGTGTACATTCCTCTCTTACCTATAA
Bsu-tyrA-F	TTATAGGTAAGAGAGGAATGTACACATGAATCAAATGAAAGATACAA TAT
Bsu-tyrA-R	AATCCGTCCTCTCTGCTCTTTCAATCAGCATAAAAAGTTTCATAT
TamyL-*Bsu-tyrA*-F	ATATGAAACTTTTTATGCTGATTGAAAGAGCAGAGAGGACGGATT
TamyL-*Bsu-tyrA*-R	GC**TCTAGA**CGCAATAATGCCGTCG
P43-*Cgb-tyrA*-F	CG**GAATTC**TGATAGGTGGTATGTTTTCGC
P43-*Cgb-tyrA*-R	ACCTGACAAGTGTGGCACATGTGTACATTCCTCTCTTACCTATAA
Cgb-tyrA-F	TTATAGGTAAGAGAGGAATGTACACATGTGCCACACTTGTCAGGT
Cgb-tyrA-R	AATCCGTCCTCTCTGCTCTTCTAGAACACTTCGATCCGTGC
TamyL-*Cgb-tyrA*-F	GCACGGATCGAAGTGTTCTAGAAGAGCAGAGAGGACGGATT
TamyL-*Cgb-tyrA*-R	GC**TCTAGA**CGCAATAATGCCGTCG
P43-*Btb-tyrA*-F	CG**GGATCC**TGATAGGTGGTATGTTTTCGC
P43-*Btb-tyrA*-R	CAATTAATACTACCTTTTTACGCATGTGTACATTCCTCTCTTACCTATA A
Btb-tyrA-F	TTATAGGTAAGAGAGGAATGTACACATGCGTAAAAAGGTAGTATTAA TTG
Btb-tyrA-R	AATCCGTCCTCTCTGCTCTTTTAAATTGTTTCATAAGTTTGGTAT
TamyL-*Btb-tyrA*-F	ATACCAAACTTATGAAACAATTTAAAAGAGCAGAGAGGACGGATT

TamyL-*Btb-tyrA*-R	GC**TCTAGA**CGCAATAATGCCGTCG
P43-*Bpum-tyrA*-F	CG**GGATCC**TGATAGGTGGTATGTTTTCGC
P43-*Bpum-tyrA*-R	GGTTTCGTTTGCTTCATTCATGTGTACATTCCTCTCTTACCTATAA
Bpum-tyrA-F	TTATAGGTAAGAGAGGAATGTACACATGAATGAAGCAAACGAAACC
Bpum-tyrA-R	AATCCGTCCTCTCTGCTCTTTCAATCAGCATAAAATGTATCGTA
TamyL-*Bpum-tyrA*-F	TACGATACATTTTATGCTGATTGAAAGAGCAGAGAGGACGGATT
TamyL-*Bpum-tyrA*-R	GC**TCTAGA**CGCAATAATGCCGTCG
P43-*Sce-tyrA*-F	CG**GGATCC**TGATAGGTGGTATGTTTTCGC
P43-*Sce-tyrA*-R	CTCAATCTTATCCTCTGATACCATGTGTACATTCCTCTCTTACCTATAA
Sce-tyrA-F	TTATAGGTAAGAGAGGAATGTACACATGGTATCAGAGGATAAGATTGAG
Sce-tyrA-R	AATCCGTCCTCTCTGCTCTTTTATGTATTTCTTTTTTCAGCGG
TamyL-*Sce-tyrA*-F	CCGCTGAAAAAAGAAATACATAAAAGAGCAGAGAGGACGGATT
TamyL-*Sce-tyrA*-R	GC**TCTAGA**CGCAATAATGCCGTCG
P43-*Bcoa-tyrA*-F	CG**GGATCC**TGATAGGTGGTATGTTTTCGC
P43-*Bcoa-tyrA*-R	ATCACAAACACATTTCCTTTCATGTGTACATTCCTCTCTTACCTATAA
Bcoa-tyrA-F	TTATAGGTAAGAGAGGAATGTACACATGAAAGGAAATGTGTTTGTGAT
Bcoa-tyrA-R	AATCCGTCCTCTCTGCTCTTCTATTGGATATAGACTTCATGCG
TamyL-*Bcoa-tyrA*-F	CGCATGAAGTCTATATCCAATAGAAGAGCAGAGAGGACGGATT
TamyL-*Bcoa-tyrA*-R	GC**TCTAGA**CGCAATAATGCCGTCG
P43-*Lpt-tyrA*-F	CG**GGATCC**TGATAGGTGGTATGTTTTCGC
P43-*Lpt-tyrA*-R	ACCTTTGATCAATACAGTTGTCATGTGTACATTCCTCTCTTACCTATAA
Lpt-tyrA-F	TTATAGGTAAGAGAGGAATGTACACATGACAACTGTATTGATCAAAGGT
Lpt-tyrA-R	AATCCGTCCTCTCTGCTCTTTTAATTCCTCCTTACAATCTGATAA
TamyL-*Lpt-tyrA*-F	TTATCAGATTGTAAGGAGGAATTAAAAGAGCAGAGAGGACGGATT
TamyL-*Lpt-tyrA*-R	GC**TCTAGA**CGCAATAATGCCGTCG
P43-*Bce-tyrA*-F	CG**GGATCC**TGATAGGTGGTATGTTTTCGC
P43-*Bce-tyrA*-R	CAATTAATACTACCTTTTTACGCATGTGTACATTCCTCTCTTACCTATAA
Bce-tyrA-F	TTATAGGTAAGAGAGGAATGTACACATGCGTAAAAAGGTAGTATTAATTG
Bce-tyrA-R	AATCCGTCCTCTCTGCTCTTTTAAATTGTTTCATAAGTTTGGTAT
TamyL-*Bce-tyrA*-F	ATACCAAACTTATGAAACAATTTAAAAGAGCAGAGAGGACGGATT
TamyL-*Bce-tyrA*-R	GC**TCTAGA**CGCAATAATGCCGTCG
P43-*Bmh*1-*tyrA*-F	CG**GGATCC**TGATAGGTGGTATGTTTTCGC
P43-*Bmh*1-*tyrA*-R	CTATAACAAAAACATTTTCTTTCACGTGTACATTCCTCTCTTACCTATAA

*Bmh*1-*tyrA*-F	TTATAGGTAAGAGAGGAATGTACACGTGAAAGAAAATGTTTTTGTTATAG
*Bmh*1-*tyrA*-R	AATCCGTCCTCTCTGCTCTTTTAAGAAATAAACGTCTGATATTGA
TamyL-*Bmh*1-*tyrA*-F	TCAATATCAGACGTTTATTTCTTAAAAGAGCAGAGAGGACGGATT
TamyL-*Bmh*1-*tyrA*-R	GC**TCTAGA**CGCAATAATGCCGTCG
P43-*Bmh*2-*tyrA*-F	CG**GGATCC**TGATAGGTGGTATGTTTTCGC
P43-*Bmh*2-*tyrA*-R	GCCGAAAATAGCAATCTTCATGTGTACATTCCTCTCTTACCTATAA
*Bmh*2-*tyrA*-F	TTATAGGTAAGAGAGGAATGTACACATGAAGATTGCTATTTTCGGC
*Bmh*2-*tyrA*-R	AATCCGTCCTCTCTGCTCTTTCACACCACCACTTTATAGCC
TamyL-*Bmh*2-*tyrA*-F	GGCTATAAAGTGGTGGTGTGAAAGAGCAGAGAGGACGGATT
TamyL-*Bmh*2-*tyrA*-R	GC**TCTAGA**CGCAATAATGCCGTCG
P43-*Zmc*-*tyrA*-F	CG**GGATCC**TGATAGGTGGTATGTTTTCGC
P43-*Zmc*-*tyrA*-R	CAATATGCTTAAAGACGGTCACGTGTACATTCCTCTCTTACCTATAA
Zmc-*tyrA*-F	TTATAGGTAAGAGAGGAATGTACACGTGACCGTCTTTAAGCATATTG
Zmc-*tyrA*-R	AATCCGTCCTCTCTGCTCTTTTAAGGGTGAATATCGTGGTCT
TamyL-*Zmc*-*tyrA*-F	AGACCACGATATTCACCCTTAAAAGAGCAGAGAGGACGGATT
TamyL-*Zmc*-*tyrA*-R	GC**TCTAGA**CGCAATAATGCCGTCG
P43-*Pap*-*tyrA*-F	CG**GGATCC**TGATAGGTGGTATGTTTTCGC
P43-*Pap*-*tyrA*-R	CGGCGTGACCTCAGTCATGTGTACATTCCTCTCTTACCTATAA
Pap-*tyrA*-F	TTATAGGTAAGAGAGGAATGTACACATGACTGAGGTCACGCCG
Pap-*tyrA*-R	AATCCGTCCTCTCTGCTCTTTCAATTGTTCTCCACGGC
TamyL-*Pap*-*tyrA*-F	GCCGTGGAGAACAATTGAAAGAGCAGAGAGGACGGATT
TamyL-*Pap*-*tyrA*-R	GC**TCTAGA**CGCAATAATGCCGTCG
P*srfA*-*Bao*-*tyrA*-F	CG**GGATCC**GACGCTCTTCGCAAGGGT
P*srfA*-*Bao*-*tyrA*-R	CAAGCAATATTGTGTCCTTCATATTGTCATACCTCCCCTAATCTT
Bao-*tyrA*-P*srfA*-F	AAGATTAGGGGAGGTATGACAATATGAAGGACACAATATTGCTTG
Bao-*tyrA*-P*srfA*-R	AATCCGTCCTCTCTGCTCTTTCAGTCAGCGTAAAATGTTTCA
TamyL-P*srfA*-*Bao*-*tyrA*-F	TGAAACATTTTACGCTGACTGAAAGAGCAGAGAGGACGGATT
TamyL-P*srfA*-*Bao*-tyrA-R	GC**TCTAGA**CGCAATAATGCCGTCG
P*ytzE*-*Bao*-*tyrA*-F	CG**GGATCC**ATCTTGACAATGCCGCTGA
P*ytzE*-*Bao*-*tyrA*-R	CAAGCAATATTGTGTCCTTCATACAACCACTCCCTATCGTATGA
Bao-*tyrA*-P*ytzE*-F	TCATACGATAGGGAGTGGTTGTATGAAGGACACAATATTGCTTG
Bao-*tyrA*-P*ytzE*-R	AATCCGTCCTCTCTGCTCTTTCAGTCAGCGTAAAATGTTTCA
TamyL-P*ytzE*-*Bao*-*tyrA*-F:	TGAAACATTTTACGCTGACTGAAAGAGCAGAGAGGACGGATT
TamyL-P*ytzE*-*Bao*-*tyrA*-R	GC**TCTAGA**CGCAATAATGCCGTCG
P*ylB*-*Bao*-*tyrA*-F	CG**GGATCC**AGCCGGATTCCTTCAGACT

P*ylB*-*Bao*-*tyrA*-R	CAAGCAATATTGTGTCCTTCATACAAATCTCCCCCTTTGTTG
Bao-*tyrA*-P*ylB*-F	CAACAAAGGGGGAGATTTGTATGAAGGACACAATATTGCTTG
Bao-*tyrA*-P*ylB*-R	AATCCGTCCTCTCTGCTCTTTCAGTCAGCGTAAAATGTTTCA
TamyL-P*ylB*-*Bao*-*tyrA*-F	TGAAACATTTTACGCTGACTGAAAGAGCAGAGAGGACGGATT
TamyL-P*ylB*-*Bao*-*tyrA*-R	GC**TCTAGA**CGCAATAATGCCGTCG
P*bay*-*Bao*-*tyrA*-F	CG**GGATCC**CCTGCGATTTCGGCGAGA
P*bay*-*Bao*-*tyrA*-R	CAAGCAATATTGTGTCCTTCATACAAATCTCCCCCTTTGTTG
Bao-*tyrA*-P*bay*-F	CAACAAAGGGGGAGATTTGTATGAAGGACACAATATTGCTTG
Bao-*tyrA*-P*bay*-R	AATCCGTCCTCTCTGCTCTTTCAGTCAGCGTAAAATGTTTCA
TamyL-P*bay*-*Bao*-*tyrA*-F	TGAAACATTTTACGCTGACTGAAAGAGCAGAGAGGACGGATT
TamyL-P*bay*-*Bao*-*tyrA*-R	GC**TCTAGA**CGCAATAATGCCGTCG
P*ykzA*-*Bao*-*tyrA*-F	CG**GGATCC**GAAATATTGATGTGACACTTGAAGT
P*ykzA*-*Bao*-*tyrA*-R	CAAGCAATATTGTGTCCTTCATATTTGTATCTAGTTGTTATATTTCC
Bao-*tyrA*-P*ykzA*-F	GGAAATATAACAACTAGATACAAATATGAAGGACACAATATTGCTTG
Bao-*tyrA*-P*ykzA*-R	AATCCGTCCTCTCTGCTCTTTCAGTCAGCGTAAAATGTTTCA
TamyL-P*ykzA*-*Bao*-*tyrA*-F	TGAAACATTTTACGCTGACTGAAAGAGCAGAGAGGACGGATT
TamyL-P*ykzA*-*Bao*-*tyrA*-R	GC**TCTAGA**CGCAATAATGCCGTCG
P*ykzA*+PRBS6-*Bao*-*tyrA*-F	CG**GGATCC**GAAATATTGATGTGACACTTGAAGT
P*ykzA*+PRBS6-*Bao*-*tyrA*-R	CAAGCAATATTGTGTCCTTCATTGATCCTTCCTCCTTTAGATCT
Bao-*tyrA*-P*ykzA*+PRBS6-F	AGATCTAAAGGAGGAAGGATCAATGAAGGACACAATATTGCTTG
Bao-*tyrA*-P*ykzA*+PRBS6-R	AATCCGTCCTCTCTGCTCTTTCAGTCAGCGTAAAATGTTTCA
TamyL-P*ykzA*+PRBS6-*Bao*-*tyrA*-F	TGAAACATTTTACGCTGACTGAAAGAGCAGAGAGGACGGATT
TamyL-P*ykzA*+PRBS6-*Bao*-*tyrA*-R	GC**TCTAGA**CGCAATAATGCCGTCG
P*mmgA*-*Bao*-*tyrA*-F	CG**GGATCC**GATTAAGGTAAGGCCGTATACAG
P*mmgA*-*Bao*-*tyrA*-R	CAAGCAATATTGTGTCCTTCATGGTTTCACCTCATTCAGAAGATA
Bao-*tyrA*-P*mmgA*-F	TATCTTCTGAATGAGGTGAAACCATGAAGGACACAATATTGCTTG
Bao-*tyrA*-P*mmgA*-R	AATCCGTCCTCTCTGCTCTTTCAGTCAGCGTAAAATGTTTCA
TamyL-P*mmgA*-*Bao*-*tyrA*-F	TGAAACATTTTACGCTGACTGAAAGAGCAGAGAGGACGGATT
TamyL-P*mmgA*-*Bao*-*tyrA*-R	GC**TCTAGA**CGCAATAATGCCGTCG
P*abrB*-*Bao*-*tyrA*-F	CG**GGATCC**CTTGAAACCTCCTAATCAGAATG

P*abrB*-*Bao*-*tyrA*-R	CAAGCAATATTGTGTCCTTCATTCTCCTCCCAAGAGATACTTATT
Bao-*tyrA*-P*abrB*-F	AATAAGTATCTCTTGGGAGGAGAATGAAGGACACAATATTGCTTG
Bao-*tyrA*-P*abrB*-R	AATCCGTCCTCTCTGCTCTTTCAGTCAGCGTAAAATGTTTCA
TamyL-P*abrB*-*Bao*-*tyrA*-F	TGAAACATTTTACGCTGACTGAAAGAGCAGAGAGGACGGATT
TamyL-P*abrB*-*Bao*-*tyrA*-R	GC**TCTAGA**CGCAATAATGCCGTCG
P*bacA*-*Bao*-*tyrA*-F	CG**GGATCC**CCTGCGATTTCGGCGAGA
P*bacA*-*Bao*-*tyrA*-R	CAAGCAATATTGTGTCCTTCATATAAAAATTCTCCTTTTTGAT
Bao-*tyrA*-P*bacA*-F	ATCAAAAAGGAGAATTTTTATATGAAGGACACAATATTGCTTG
Bao-*tyrA*-P*bacA*-R	AATCCGTCCTCTCTGCTCTTTCAGTCAGCGTAAAATGTTTCA
TamyL-P*bacA*-*Bao*-*tyrA*-F	TGAAACATTTTACGCTGACTGAAAGAGCAGAGAGGACGGATT
TamyL-P*bacA*-*Bao*-*tyrA*-R	GC**TCTAGA**CGCAATAATGCCGTCG
PUTR12-*Bao*-*tyrA*-F	CG**GGATCC**TGATAGGTGGTATGTTTTCGC
PUTR12-*Bao*-*tyrA*-R	CAAGCAATATTGTGTCCTTCATTATATATTCCTCCTTTCTAATATAC
Bao-*tyrA*-PUTR12-F	GTATATTAGAAAGGAGGAATATATAATGAAGGACACAATATTGCTTG
Bao-*tyrA*-PUTR12-R	AATCCGTCCTCTCTGCTCTTTCAGTCAGCGTAAAATGTTTCA
TamyL-PUTR12-*Bao*-*tyrA*-F	TGAAACATTTTACGCTGACTGAAAGAGCAGAGAGGACGGATT
TamyL-PUTR12-*Bao*-*tyrA*-R	GC**TCTAGA**CGCAATAATGCCGTCG
P43-*ylB*-*Bao*-*tyrA*-F	CG**GGATCC**TGATAGGTGGTATGTTTTCGC
P43-*ylB*-*Bao*-*tyrA*-R	CAAGCAATATTGTGTCCTTCATACAAATCTCCCCCTTTGTTG
Bao-*tyrA*-P43-*ylB*-F	CAACAAAGGGGGAGATTTGTATGAAGGACACAATATTGCTTG
Bao-*tyrA*-P43-*ylB*-R	AATCCGTCCTCTCTGCTCTTTCAGTCAGCGTAAAATGTTTCA
TamyL-P43-*ylB*-*Bao*-*tyrA*-F	TGAAACATTTTACGCTGACTGAAAGAGCAGAGAGGACGGATT
TamyL-P43-*ylB*-*Bao*-*tyrA*-R	GC**TCTAGA**CGCAATAATGCCGTCG
PR5-*Bao*-*tyrA*-F	CG**GGATCC**TGATAGGTGGTATGTTTTCGC
PR5-*Bao*-*tyrA*-R	CAAGCAATATTGTGTCCTTCATTGATCCTTCCTCCTTTAGATCTG
Bao-*tyrA*-PR5-F	CAGATCTAAAGGAGGAAGGATCAATGAAGGACACAATATTGCTTG
Bao-*tyrA*-PR5-R	AATCCGTCCTCTCTGCTCTTTCAGTCAGCGTAAAATGTTTCA
TamyL-PR5-*Bao*-*tyrA*-F	TGAAACATTTTACGCTGACTGAAAGAGCAGAGAGGACGGATT
TamyL-PR5-*Bao*-*tyrA*-R	GC**TCTAGA**CGCAATAATGCCGTCG
PRBS6-*Bao*-*tyrA*-F	CG**GGATCC**TGATAGGTGGTATGTTTTCGC

PRBS6-*Bao*-*tyrA*-R	CAAGCAATATTGTGTCCTTCATTGATCCTTCCTCCTTTAGA
Bao-*tyrA*-PRBS6-F	TCTAAAGGAGGAAGGATCAATGAAGGACACAATATTGCTTG
Bao-*tyrA*-PRBS6-R	AATCCGTCCTCTCTGCTCTTTCAGTCAGCGTAAAATGTTTCA
TamyL-PRBS6-*Bao*-*tyrA*-F	TGAAACATTTTACGCTGACTGAAAGAGCAGAGAGGACGGATT
TamyL-PRBS6-*Bao*-*tyrA*-R	GC**TCTAGA**CGCAATAATGCCGTCG
P43-UTR1-*Bao*-*tyrA*-R1	GTGTACCTTCCTCTCTGACTTATAATGGTACCGCTATCACTTTATATT
P43-UTR1-*Bao*-*tyrA*-R2	CAAGCAATATTGTGTCCTTCATGTGTACCTTCCTCTCTGACTTATAA
Bao-*tyrA*-UTR1-F	TTATAAGTCAGAGAGGAAGGTACACATGAAGGACACAATATTGCTTG
Bao-*tyrA*-UTR1-R	AATCCGTCCTCTCTGCTCTTTCAGTCAGCGTAAAATGTTTCA
TamyL-UTR1-*Bao*-*tyrA*-F	TGAAACATTTTACGCTGACTGAAAGAGCAGAGAGGACGGATT
TamyL-UTR1-*Bao*-*tyrA*-R	GC**TCTAGA**CGCAATAATGCCGTCG
P43-UTR2-*Bao*-*tyrA*-R1	GTGTACCTTCCTCTCTTACCTATAATGGTACCGCTATCACTTTATATT
P43-UTR2-*Bao*-*tyrA*-R2	CAAGCAATATTGTGTCCTTCATGTGTACCTTCCTCTCTTACCTATAA
Bao-*tyrA*-UTR2-F	TTATAGGTAAGAGAGGAAGGTACACATGAAGGACACAATATTGCTTG
Bao-*tyrA*-UTR2-R	AATCCGTCCTCTCTGCTCTTTCAGTCAGCGTAAAATGTTTCA
TamyL-UTR2-*Bao*-*tyrA*-F	TGAAACATTTTACGCTGACTGAAAGAGCAGAGAGGACGGATT
TamyL-UTR2-*Bao*-*tyrA*-R	GC**TCTAGA**CGCAATAATGCCGTCG
P43-UTR3-*Bao*-*tyrA*-R1	GCGTACCTTCCTCTCTTACTTATAATGGTACCGCTATCACTTTATATT
P43-UTR3-*Bao*-*tyrA*-R2	CAAGCAATATTGTGTCCTTCATGCGTACCTTCCTCTCTTACTTATAA
Bao-*tyrA*-UTR3-F	TTATAAGTAAGAGAGGAAGGTACGCATGAAGGACACAATATTGCTTG
Bao-*tyrA*-UTR3-R	AATCCGTCCTCTCTGCTCTTTCAGTCAGCGTAAAATGTTTCA
TamyL-UTR3-*Bao*-*tyrA*-F	TGAAACATTTTACGCTGACTGAAAGAGCAGAGAGGACGGATT
TamyL-UTR3-*Bao*-*tyrA*-R	GC**TCTAGA**CGCAATAATGCCGTCG
P43-UTR4-*Bao*-*tyrA*-R1	GTGTACCTTCCTCTCTAACTCATAATGGTACCGCTATCACTTTATATT
P43-UTR4-*Bao*-*tyrA*-R2	CAAGCAATATTGTGTCCTTCATGTGTACCTTCCTCTCTAACTCATAA
Bao-*tyrA*-UTR4-F	TTATGAGTTAGAGAGGAAGGTACACATGAAGGACACAATATTGCTTG
Bao-*tyrA*-UTR4-R	AATCCGTCCTCTCTGCTCTTTCAGTCAGCGTAAAATGTTTCA

TamyL-UTR4-*Bao-tyrA*-F	TGAAACATTTTACGCTGACTGAAAGAGCAGAGAGGACGGATT
TamyL-UTR4-*Bao-tyrA*-R	GC**TCTAGA**CGCAATAATGCCGTCG
P43-UTR5-*Bao-tyrA*-R1	GCGTACCTTCCTCTCTAACCTATAATGGTACCGCTATCACTTTATATT
P43-UTR5-*Bao-tyrA*-R2	CAAGCAATATTGTGTCCTTCATGCGTACCTTCCTCTCTAACCTATA
Bao-tyrA-UTR5-F	TATAGGTTAGAGAGGAAGGTACGCATGAAGGACACAATATTGCTTG
Bao-tyrA-UTR5-R	AATCCGTCCTCTCTGCTCTTTCAGTCAGCGTAAAATGTTTCA
TamyL-UTR5-*Bao-tyrA*-F	TGAAACATTTTACGCTGACTGAAAGAGCAGAGAGGACGGATT
TamyL-UTR5-*Bao-tyrA*-R	GC**TCTAGA**CGCAATAATGCCGTCG
P43-UTR6-*Bao-tyrA*-R1	GCGTACCTTCCTCTCTGACTCATAATGGTACCGCTATCACTTTATATT
P43-UTR6-*Bao-tyrA*-R2	CAAGCAATATTGTGTCCTTCATGCGTACCTTCCTCTCTGACTC
Bao-tyrA-UTR6-F	GAGTCAGAGAGGAAGGTACGCATGAAGGACACAATATTGCTTG
Bao-tyrA-UTR6-R	AATCCGTCCTCTCTGCTCTTTCAGTCAGCGTAAAATGTTTCA
TamyL-UTR6-*Bao-tyrA*-F	TGAAACATTTTACGCTGACTGAAAGAGCAGAGAGGACGGATT
TamyL-UTR6-*Bao-tyrA*-R	GC**TCTAGA**CGCAATAATGCCGTCG

Note: Restriction sites highlight in bold.
